# Pinxian Wang: no place for a ‘gold rush’ in the exploration of the deep sea

**DOI:** 10.1093/nsr/nwac260

**Published:** 2022-11-17

**Authors:** Weijie Zhao, Zhonghe Zhou

**Affiliations:** Zhonghe Zhou is a professor at the CAS Institute of Vertebrate Paleontology and Paleoanthropology, and an Associate Editor-in-Chief of NSR; Weijie Zhao is an NSR news editor based in Beijing; Zhonghe Zhou is a professor at the CAS Institute of Vertebrate Paleontology and Paleoanthropology, and an Associate Editor-in-Chief of NSR; Weijie Zhao is an NSR news editor based in Beijing

## Abstract

Prof. Pinxian Wang (汪品先) of Tongji University, born in 1936, is one of the leading deep-sea scientists in China. As the first co-chief scientist of Chinese nationality in the international Ocean Drilling Program (ODP), he co-led ODP Leg 184 in 1999; and in 2018, at 82 years old, he dived to a depth of 1400 meters in the South China Sea (SCS) in China's manned submersible ‘Deep Sea Warrior’. In recent years, he has also become a star of science popularization in China—he has more than 1.7 million followers on China's video-sharing platform *bilibili*, and his popular science book Deep Sea in Simple Words is a best seller.

In September 2022, Prof. Wang was interviewed by *NSR*’s Associate Editor-in-Chief, Zhonghe Zhou. In this interview, Prof. Wang summarized his six-decade-long scientific career, talked about the past and future of China's deep-sea research efforts, and explained his opinion on how to promote scientific culture in China.

## 62-year scientific career: two areas promoted, two hypotheses proposed

**Zhou:** You have been a researcher for more than 60 years. What are your major achievements?

**Wang:** I studied geology at Moscow University in the former Soviet Union, and returned to China in 1960. From then on, I have been working in marine science for 62 years. In the first 30 years, I did some work on the shallow sea, but the achievements were limited, which is to say, almost all the work that I am satisfied with came out in the last 30 years—after I became 60 years old.

In my own opinion, my major achievements are: I promoted two areas of research in China, and proposed two scientific hypotheses. The two research areas are deep-sea research and Earth system science. The two hypotheses are the low latitude forcing of climate change and, together with colleagues, the new idea of the formation of the SCS.

**Zhou:** You were selected for the Chinese Academy of Sciences (CAS) in 1991, but according to what you just said, your major work was done after that?

**Wang:** That's right. In the early years, I did some work on the paleontology of the shallow sea, but I was not a good paleontologist—I had worked on several groups of microfossils, such as foraminifer, ostracoda and coccoliths, along with macrofossils including molluscs and graptolites, but I did not work deeply on any of them. Then in the 1980s, I was engaged in sea-level changes, namely the Quaternary transgression and regression. I suppose I was selected for CAS mostly because of these works, but actually, I had focused on only a rather narrow research direction in the area of environmental changes.

**Zhou:** Let's move to the major achievements that you have mentioned. The first is that you promoted China's deep-sea research.

**Figure 1:**
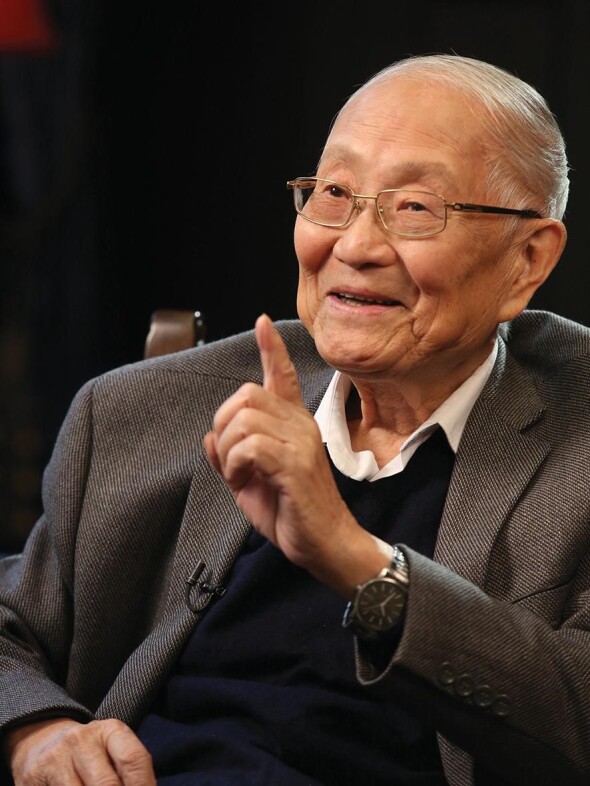
Prof. Pinxian Wang on a TV show *(courtesy of Prof. Wang)*.

**Wang:** Yes. Deep-sea research had never been a hot field in China, but the situation has changed remarkably in the last few decades. Over the years, we tried to call attention to the new field in our country, and our efforts were not in vain.

First, I promoted China's participation in the international ODP. China's first attempt to join ODP was made in 1985, but did not succeed. It was in 1997 when we finally became a member of ODP. In 1998, China officially joined ODP, and in 1999, I was one of the two chief scientists of ODP Leg 184, a drilling expedition in the SCS. From then on, there have been several more ODP chief scientists from China.

Second, we founded the first State Key Laboratory of Marine Geology in the country. This lab was established at the Tongji University in the 1990s, and then upgraded into a State Key Laboratory in 2005.

Third, we promoted a big program to build China's Seafloor Observatory Network, as one of the large-scale scientific facilities in China. The application of this billion-yuan-level program was started in 2012. Now it has been approved and is supported by the National Development and Reform Commission as well as the Shanghai government.

Fourth, we initiated a series of International Conferences on Asian Marine Geology, which started in 1988 and was held in different Asian countries every three years until recently. It is a well-organized international conference series and has greatly promoted the study of marine geology in Asia.

Lastly, we accomplished the SCS Deep Research Program. Lasting from 2011 to 2018, the program was the largest ever basic marine science endeavor, with some 30 laboratories involved across the country. To reach its scientific goals, we organized three international deep-drilling expeditions—each lasting two months—as well as three diving cruises, and deployed numerous mooring arrays for long-term observation of deep-sea processes.

Looking back on the past few decades, I really appreciate how the progress of deep-sea science in China was instigated by ourselves.

**Zhou:** These are great contributions. What about your efforts in promoting Earth system science?

**Wang:** The concept of the Earth system has been proposed for a long time, but there were many misunderstandings about it in our country: some considered Earth system science as a new term of physical geography, and others equated it with remote sensing. What I did was to clarify the concept: the Earth system includes all the spheres of the Earth, and as a science it especially emphasizes their mutual interactions. We wrote corresponding papers and textbooks, organized conferences, and currently we are working on a strategic plan of its development in China.

**Zhou:** Let's move to your two major scientific opinions. The first is the low latitude forcing concept of climate change.

**Wang:** Yes. Actually, my two major scientific hypotheses were both rooted in ocean drilling practices. In the 1999 ODP expedition, we recovered a continuous sequence of deep-sea sediments of the last 20 million years for the first time in the region, which enabled our research on climate change, and the results of the analyses revealed low latitude forcing of climate changes. Traditionally, scientists have believed that Earth's climate change is mainly driven by the glacial cycle of the Arctic, but our new data indicated a major forcing role of the low latitudes where the solar insolation is focused. We linked the northern pole to a switchboard, and the low latitude area to an engine, and the switchboard alone cannot drive climate change without the engine.

Since the new idea challenges the traditional wisdom, the publication of our ideas has never been smooth. Quite often, several rounds of revision and rebuttal were needed to convince the reviewer(s) before the final publication.

**Zhou:** The other achievement you mentioned is the origin mechanism of the SCS. I think it's one of your favorite ideas.

**Wang:** The opening of marine basins is one of the fundamental issues in geology, and related research has been constantly focusing on the Atlantic Ocean. In 2017, two successive cruises, IODP Expeditions 367 and 368, were implemented in the SCS to test the applicability of the Atlantic model of non-volcanic passive margin to the SCS. The key lies in serpentinized mantle, which weakens the lithosphere. Contrary to expectations, serpentinite was not found in the expeditions. Instead, we coved many cores of basalt, which disproved the original assumption of its non-volcanic origin.

Thus, we announced that ‘the SCS is not a mini Atlantic’ and the opening mechanisms of the two basins are completely different. Simply speaking, the origin of the Atlantic is the result of the collapse of a supercontinent, while the origin of the SCS is a by-product of the subduction of a superocean, or we can say, of a ‘divergence (opening) in convergence (subduction)’. Potentially, the SCS discovery will broaden our understanding of plate tectonics. In the classic tectonic theory, we care mainly about the formation and disruption of supercontinents. But the new SCS origin mechanism suggests that the superoceans also have their own stories and hence deserve further study.

**Zhou:** Has this idea been recognized by international scientists?

**Wang:** We have published this idea in NSR, but the story is not limited to the SCS alone, as our new concept involves the whole system of the West Pacific marginal seas. Obviously, it will require many years of systematic research before the hypothesis becomes mature.

The origin of the Atlantic is the result of the collapse of a supercontinent, while the origin of the South China Sea is a by-product of the subduction of a superocean.

—Pinxian Wang

## Deep-sea exploration: the three ‘deeps’

**Zhou:** Why have so many scientists been interested in the deep sea in recent years?

**Wang:** In the early years, there were not so many Chinese scientists interested in the deep sea. In the 1990s, I gave a talk on a forum called ‘Tomorrow's Science’ held by Shanghai's Association for Science and Technology. I talked about the deep sea, and the chair commented, ‘What Pinxian Wang talked about is not tomorrow's science; it's the science of the day after tomorrow.’ So, seeing deep-sea research receiving more and more recognition in recent years, I am quite pleased.

Worldwide, humanity’s exploration of the oceans started in the coastal shallow sea. This ocean research first focused on the development of the industries of fishing, salt production and sea transportation; and then slowly transferred to the exploration of the deep sea and seafloor, in order to exploit deep-sea resources. In summary, in the 16th century, humans entered the ocean in the horizontal dimension, then starting from the late 20th century, we began to enter the deep sea and explore the ocean in the vertical dimension.

**Zhou:** So, humanity’s first explorations of the deep sea were aimed at exploiting its resources?

**Wang:** That's right. The first resource that drew a lot of attention were manganese nodules. In the 1960s, some US scientists claimed that these nodules grew very fast in the deep sea, and were almost inexhaustible as a resource. But now we know that this is not true. Manganese nodules grow extremely slowly, and to date, they have not been commercially mined. Instead, deep-sea oil and gas have become the pillar industry in maritime economy, and gas hydrates have also drawn much attention.

These stories remind us that there is no place for a ‘gold rush’ in the exploration of the deep sea, because most deep-sea resources are formed by slow processes. The growth of some deep-sea corals can be as slow as several microns a year, which means that if we collect and destroy these corals, it would take thousands of years for them to recover.

Moreover, if we simply use the investigation results of several sampling points to predict the situation of the entire global ocean, it will very possibly lead to an overestimation of the amount of deep-sea resources and therefore unpleasant consequences.

There is no place for a ‘gold rush’ in the exploration of the deep sea, because most deep-sea resources are formed by slow processes.

—Pinxian Wang

**Zhou:** What are the major approaches for exploring the deep sea?

**Wang:** The most direct approaches for exploring the deep sea are the three ‘deeps’: deep diving, deep drilling and deep networking for observations.

Historically, deep diving came first. In the 1930s, the US started to develop deep-diving technologies, and now, many countries, including China, are able to build advanced deep-diving submersibles. It's good that China is paying attention to manned submersibles, but it should not be at the cost of unmanned deep-diving robots. Manned submersibles are attractive to the media and the public, but actually, robots can make many more contributions to scientific research, and at a much lower cost.

**Zhou:** You dived into the deep sea three times. With regard to these experiences, is manned deep diving valuable for scientific research?

**Wang:** It depends on who is diving. It is useful if the people diving down are real scientists, who can evaluate the scientific value of the things they see, and decide which samples to collect for further analysis. But considering that the cost of manned deep diving is very high, the best choice for the future is to combine manned and unmanned deep-diving technologies, and make full use of robots.

Again, it's good that our media is interested in, and actively reports, scientific diving, but the focus was on technology more than science. The major goals were to dive deeper. Now the deepest site on Earth has been reached, our media's interest should move to science, to report how we solve real scientific problems.

**Zhou:** What about deep drilling and deep networking?

**Wang:** Deep drilling started in the 1960s. The US was the first country to develop these technologies, and is still the leading force. Deep drilling has basically been promoted by international cooperation from the very beginning.

Deep networking, or deep sea observation networking, began in the late 1990s, and its fast development has only started since the turn of the century. Different from deep diving and deep drilling, which both investigate one site at a time, deep networking aims to cover large ocean areas and to observe biological, geological, chemical and physical processes simultaneously. Currently, the US, Canada and Japan are leading this field, and China and Europe are also catching up. The building of deep networking is a comprehensive task, which needs to deploy various kinds of sensors and other devices into the ocean, and to transmit and gather the data through wired or wireless networks. I believe that the future development of deep networking will also need international cooperation.

China is just getting started in both deep drilling and deep networking. We need to cooperate with the international community for further developments.

**Zhou:** International cooperation has been hindered by the changing international environment in recent years. What can Chinese scientists do to maintain and promote international cooperation in marine science?

**Wang:** Independence is now emphasized by many Chinese. But for marine science, international cooperation is indispensable for China's further development. Taking deep drilling as an example, I think China should cooperate with both the developed and the developing countries.

Over the decades, China has made tremendous progress in marine science and technology and benefited greatly from international cooperation, but there is still a long way to go to really reach global advanced levels. In terms of ocean drilling, China is eager to be one of the platform players; meanwhile, cooperation with developed countries is badly needed for both science and technology.

On the other hand, deep sea drilling programs have developed to a stage that needs the participation of developing countries—important locations in the open sea have been largely drilled, and further researches require drilling in the exclusive economic zones of many developing countries. How can this best go ahead? My suggestion is that these developing countries should be welcomed into ocean drilling programs. They probably do not need to invest money, as the developed countries do, but rather provide political support.

I suppose that the future international ocean drilling program will develop into a new mode. The US, Europe, Japan and China each have their own operation plans, but they have a shared science committee and maintain cooperation with each other. For China, we should have our science implementation plan and cooperate with neighboring developing countries. Our drilling priorities will be in the SCS and Western Pacific, as well as some other ocean regions.

**Zhou:** In recent years, a number of marine labs and schools of oceanography have been established in China's coastal cities and universities. What's your comment on these developments?

**Wang:** It is a rare phenomenon for a country to set up so many universities and schools of marine science and technology, and train large amounts of undergraduate students majoring in this field. In my opinion, the best way to cultivate oceanography talents is not to set up many undergraduate schools, but to encourage students of various basic disciplines, whether that's physics, chemistry, geology or biology, to engage in marine science questions in their graduate stages.

It is a good thing that so many Chinese cities and universities are interested in marine science and deep-sea research. But we should not blindly expand the educational and developmental scales. We should have a national overall plan and encourage cities and institutions to develop with different priorities, but not to do the same thing at the same time.

## Being a popular science star

**Zhou:** You are active in the fields of education and science popularization. You have more than 1.7 million followers on *bilibili*, and your undergraduate course ‘Science and Culture’ is highly praised by students. When did you begin to be interested in this work?

**Wang:** I started to promote marine science when I was asked to give lectures to government officials. These lectures received rather good evaluations. My popularization of science for kids and teenagers started with the editing of the marine volume of the PartWork *100 000 Whys*. This series of books is very famous and popular in China, and I was engaged in its new edition's editing in 2011. I spent a lot of time and effort on this book and emphasized two points at that time: first, the book should include contents about the deep sea; second, it is more important to ask high quality ‘whys’ than to answer these questions.

However, being in science popularization videos and becoming a science star on the web was not something that happened on my own initiative, but was promoted by some journalists who had interviewed me. I have accounts on TikTok, *bilibili* and some other platforms, and it was a surprise for me to have so many followers, especially on *bilibili*. The TikTok videos are very short, limited to dozens of seconds, but the videos on *bilibili* can be longer, and may be more suitable for science-related content.

**Zhou:** How does it feel to be a cyber star? Did it bother you in any sense?

**Wang:** I think it did not bother me much. Sometimes I am recognized on the street and asked to take photos with students, but it's okay for me.

Becoming a cyber star means gaining publicity. You can exchange the publicity for money or other things. For me, I want to exchange it for social impact. I hope that my opinions and suggestions can be noticed and seriously considered by more people.

**Zhou:** Popularizing science at your age is admirable, but for many younger scientists, such practices may be considered frivolous.

**Wang:** Yes, such prejudice does exist in China. But actually, teaching, research and science popularization are closely interconnected. Teaching and science popularization practices can promote and realize the social value of scientific research.

## When modern science meets traditional Chinese culture

**Zhou:** You have mentioned many times that we should build scientific ‘Chinese schools’. Why is it important?

**Wang:** There are two reasons, one from the perspective of China, and the other from the perspective of the world. From China’s perspective, our scientific research has now developed to a stage where in some particular research fields we are now able to act as one of the leading forces.

From a global perspective, scientific research is now encountering obstacles that are hard to overcome. Senior science reporter John Horgan wrote the book *The End of Science* in 1996, which said that the frame and basic theories of modern science had been completed, and that future development would just be minor repairs and supplements. But now we know that this opinion was wrong. Science is still far from its end, and has already met new obstacles.

To overcome these obstacles, I think we need a fundamental change of mind in scientific philosophy, rather than technological improvements only. Modern science has been driven by mainly analytical methods for hundreds of years. Disciplines are divided more and more narrowly, but new trends call for the opposite. Take Earth science, my own research field, as an example. In recent decades, the emergence of remote sensing, big

Western science has always been alien to Chinese culture. One of the reasons is that we want to use Western technologies without changing our traditional culture.

—Pinxian Wang

data and large-scale calculation has begun to call for synthetic and comprehensive research methods. However, will this *synthesis* be achieved by merely more and more data and bigger and bigger calculation scales? I think the answer is no. We need a fundamental pattern change, and some parts of China's traditional culture may contribute to this change. I am not sure how this change will happen, or how Chinese culture will help, but I think I can vaguely see some hints.

**Zhou:** But there has long been an opinion that traditional Chinese culture and modern science are irreconcilable in some aspects.

**Wang:** This opinion is also reasonable. Modern science was first brought into China by Western missionaries; then the Westernization Movement of the Qing Dynasty translated Western books into Chinese, sent Chinese students to Western countries, and tried to adopt Western science and technology. After that, many Chinese students returned to China from abroad and brought with them the research ideas of their Western mentors. However, all these import practices were not systematic enough, and Western science has always been alien to Chinese culture. One of the reasons is that we want to use Western technologies without changing our traditional culture. As a comparison, Japan accepted Western science and culture more systematically, and gained faster and greater scientific development.

The thoughts of many Chinese scholars are still traditional now. They still admire authority. They are used to ‘speaking for the ancient wise’, but not speaking for themselves. Unfortunately, this tradition is a killer of scientific innovation. Several years ago, I inscribed a sentence for the first-year students of Tongji University: The key to morality is faith; the key to science is doubt. Scientific research cannot be done without the spirit of suspicion.

## Retrospection and perspective

**Zhou:** What are the fortunes and misfortunes of your life?

**Wang:** My fortunes and misfortunes are not my own. They are mostly shared experiences of my generation. But I have been rather lucky throughout my life. I went to learn in the former Soviet Union in the 1950s, visited the Western countries as a member of the Petroleum-Scientists Delegation in 1978, and went to Germany as a Humboldt Research Fellow in the 1980s. Humboldt Research Fellows are generally required to be younger than 35, but they offered some Chinese scholars a special chance at that time—we were more than 40 years old when we went to Germany. These are all the best and luckiest opportunities for me to have.

I am also lucky to have promoted the new developments of China's marine science after I turned 60. So maybe it is not wise for scholars and scientists to retire at the age of 60. Many scientists at 60 years old have just become mature in thought, and are able to make great contributions.

**Zhou:** I heard that you have a personal five-year plan. Would you please introduce it?

**Wang:** My current five-year plan is a plan for 2021 to 2026, when I will be 90 years old. First, I will publish another popular science book. It is almost completed and will come out soon. A publishing house is also trying to turn my public lecture course ‘Science and Culture’ into a book. Second, I will continue with my research work in the SCS, and publish our hypotheses in both Chinese and English. Third, I think it's a responsibility of people of my generation to write down our personal experiences and thoughts, and leave these documents for future generations. It would take some time for me to do it, and I don’t know when it would be completed. People of my age always feel that time is very precious.

**Zhou:** Our last question is about the younger generation. What are the differences between the environment your generation grew up in, and that of the current young generation? What are your suggestions for the youngsters?

**Wang:** Throughout the world, people of my generation, the generation grown during the Second World War, have some shared characteristics. We care a lot about the public interest, and are willing to contribute to society without regard for personal benefit. The current young generation, who have grown up in a peaceful society, especially those who have grown up in rich families, tend to care more about what they have or do not have. With a strong ‘entitlement’ feeling, they care more about personal benefit and personal success. This is a basic difference and cannot be summarized by the word ‘generation gap’. One thing we can do is let today's young people learn more about history, so that they can view personal values and the development of the world from a wider and higher perspective.

